# Genetic relationships and introgression events between wild and cultivated grapevines (*Vitis vinifera* L.): focus on Italian Lambruscos

**DOI:** 10.1038/s41598-024-62774-w

**Published:** 2024-05-29

**Authors:** A. Schneider, P. Ruffa, G. Tumino, M. Fontana, P. Boccacci, S. Raimondi

**Affiliations:** 1 Institute for Sustainable Plant Protection, National Research Council of Italy (IPSP-CNR), Strada delle Cacce 73, 10135 Turin, Italy; 2 Giovanni Dalmasso Foundation, Largo Braccini 2, 10095 Grugliasco, Turin, Italy; 3 Department of Agricultural, Forest and Food Sciences, University of Turin (DiSAFA-UNITO), L. Braccini 2, 10095 Grugliasco, Turin, Italy; 4 Plant Breeding, Wageningen University and Research (WUR), P.O. Box 9101, 6700 HB Wageningen, The Netherlands; 5 Bagnacavallo, Italy

**Keywords:** Genetics, Plant sciences

## Abstract

Research efforts on genomic structure and ecology of wild populations of *Vitis vinifera* L. offer insights on grape domestication processes and on the assortment evolution of the cultivated forms. Attention is also paid to the origin of traditional, long-cultivated varieties, often producing renowned and valuable wines. The genetic relationships between 283 *Vitis vinifera* cultivated varieties (subsp. *sativa*) and 65 individuals from 9 populations of the *sylvestris* subspecies mainly from northern Italy were explored by means of molecular markers (27 nuclear and 4 chloroplastic microsatellites). Several episodes of contamination of the wild germplasm by the pollen of specific grape cultivars were detected, implying concern for maintaining the purity of the wild form. At the same time, events of introgression from the wild subspecies resulted playing a crucial role in the emergence of several cultivated varieties with a clear admixed genome ancestry *sativa*-*sylvestris*. These included Lambruscos originated from the flat areas crossed by the Po and Adige rivers in northern Italy, while other cultivars still called Lambrusco but typical of hilly areas did not show the same admixed genome. Historical and ecological evidences suggesting an adaptative recent post-domestication process in the origin of several Italian Lambruscos are discussed.

## Introduction

Grapevine is the fruit crop of major economic value, covering a surface of around 7.33 Mha worldwide with a production of 75 Mt of grapes mainly used for wine and fresh consumption (statistics 2020 by OIV, https://www.oiv.int/it/what-we-do/global-report?oiv).

The Eurasian species *Vitis vinifera* L. is by far the most cultivated. Besides the domesticated subspecies *vinifera* (or *sativa*), its wild relative subsp. *sylvestris* still exists in the temperate areas of Europe, northern Africa, western Asia^1^. Wild *vinifera* grapevines differ from the domesticated vines for three major traits: plant sex (they have dioecious plants while the cultivated are generally hermaphrodite, seldom female), size of bunches and berries (smaller in the wild forms), shape of the pips (more rounded and with a shorter beak in *sylvestris*)^2^. There are no breeding barriers between the two subspecies, so that gene flow can occur in both directions in any overlapping distribution areas.

Archaeological and historical records suggest grapevine domestication took place from the wild progenitor around 8000 years ago at the drawn of the Neolithic, in an area between eastern Anatolia and northern Mesopotamia^3^. Recent genomic evidences based on divergence between wild *sylvestris* and cultivated samples predate to 22,000 years ago the beginning of the domestication process^4^. According to Zhou and collaborators^5^ there was a long period of low-intensity management by humans prior purposeful cultivation in the Transcaucasia region. A recent study describing the phenomenon as a whole^6^ proposes grapevine domestication took place about 11,000 years ago in two different sites for table and wine grapes, in Western Asia and Caucasus respectively.

In addition to the relocation from a natural to a cultivated habitat close to permanent habitation^7^, domestication included the selection of elite variants, their vegetative propagation by cuttings and further improvement by the use of recombinants from natural inter-crosses. One of the key changes in this progression was the breakdown of dioecy, together with the increasing in berry number and size, sugar content and the emergence of differently coloured grapes.

With the spreading of wine culture, domesticated vines expanded westwards for several millennia from the prime (or primes) Western Asian centre (centres). Genetic studies had proved the contribution of the local *sylvestris* germplasm to the development of the cultivated European gene pool by comparing samples from the two *vinifera* subspecies^8–12^. Moreover, the identity between chlorotypes (of maternal inheritance in grapevine) from wild vines and from many local cultivars from the Iberian Peninsula, brought evidence of a secondary domestication event, or at least the introgression from local *sylvestris* into the cultivated populations^13,14^. Other additional domestication centres have been proposed according to a multi-origin hypothesis, with independent lineages developing from wild progenitors in different European areas, as reported in the review by Grassi and De Lorenzis^15^.

On the other hand, events of exchange in the opposite direction, i.e. gene flow from the cultivated compartment towards the wild populations, were also documented^14,16^. These facts, which threaten the survival of pure wild forms, must be promptly recognized. At the same time, also feral accessions, i.e. plants escaped from cultivation, must be clearly identified^17^.

Most of the traditional wine grape varieties currently cultivated arose from natural hybridizations followed by clonal propagation often going on for hundreds of years^2^. Generally, only few main genitors emerged, so that the cultivated pool results often structured in regional groups of related varieties or in larger clusters geographically distributed^8,18–25^. Because of the wide use of propagation by cuttings, only few generations of sexual reproduction occurred in the development of historical grape cultivars^26^. This was also proved by paleo-genomics studies, showing several varieties still grown today first-degree related to genotypes (likely extinct) recovered in Roman sites dating back to I-II century CE^27^.

Populations or isolated individuals of wild *vinifera*, representing what nowadays remains of a previous wide diffusion, have been inventoried and often described in most of the temperate regions from the Iberian Peninsula to northern Iran^28–32^. The observed habitats are alluvial or riparian forests along major rivers and their tributaries, more rarely colluvial areas. The interest towards the wild subspecies, today strongly threatened of extinction, lies in the effort: (a) to preserve the widest possible range of genetic diversity for breeding purpose, (b) to better understand the domestication process and the genetic basis of the major selected traits, and c) to outline the origin of some elite cultivars and their history.

Likely due to the mild climate, a great number of *sylvestris* populations were recorded also across the Italian peninsula and in the major islands^33–35^, indicating Italy as one of the possible refuge areas during the glaciations, and therefore postglacial colonization source. In Italy, in fact, there are abundant and ancient literary references to the wild grapevine, defined as *labrusca* or *lambrusca*. According to Sereni^36^, the oldest literary attestation of *labrusca* is attributed to the Latin author Virgil (70–19 BC) born in Mantua, northern Italy. Other references to wild vines followed, including the one by De Crescentiis^37^ from Bologna (in the Emilia-Romagna region) “there are wild vines, called labrusche, which grow freely on trees”. The term *lambrusques* to indicate plants of the *sylvestris* subspecies also remains in French^38^, due to the common Provençal/Paleo-Ligurian linguistic matrix^36^ geographically referable to southern France and north-western and central Italy. Indeed, it does not seem a coincidence that several grape cultivars called Lambrusco, typical of an area south of the river Po in north-central Italy, clustered in a group intermediate between the two subspecies *sylvestris* and *vinifera*^39^ or were considered primitive from an historical point of view, as a transition stage between wild grapes and more evolved cultivated varieties^40^.

The study we present here explores the genetic relationships among indigenous *sylvestris* populations and local traditional grape cultivars, including 18 named Lambrusco, whose origin from on-site domestication has been proposed but not deeply investigated yet. By means of molecular genotyping (27 nSSR loci well distributed into the genome and 4 cpSSR loci) we analysed 283 cultivated varieties mainly from northern Italy and 65 *sylvestris* individuals from 9 populations located in northern Italy and southern France. Contamination from the domesticated *vinifera* into the subspecies *sylvestris* and the contribution of the latter to the birth of several today’s historical cultivars are discussed. An hypothesis about the origin of several Lambrusco varieties denying their ancient ancestry is presented.

## Results

**Figure 1:**
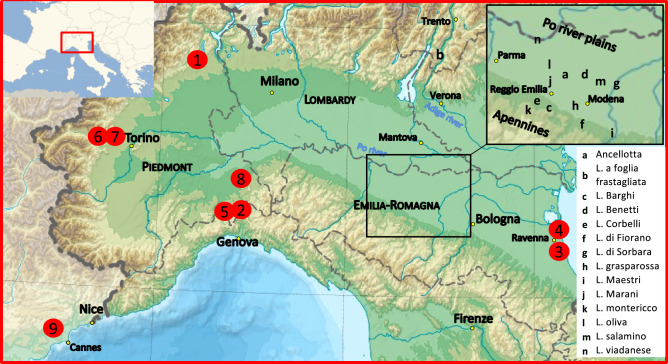
Geographical distribution of the wild examined grapevine populations (red circles and number according to Supplementary Table S3) and of the traditional growing area of Lambrusco varieties in the Italian region of Emilia-Romagna and Trentino (black letters). Image modified from Blank topographic map from Wikimedia Commons. © Eric Gaba for Wikimedia Commons. Licensed under CC BY-SA 3.0 license (https://commons.wikimedia.org/wiki/File:Italy_topographic_map-blank.svg).

From the 348 accessions examined in this study (283 belonging to cultivated and 65 to wild grapevines, Supplementary Table S1), 348 unique nSSR profiles were obtained (Supplementary Table S2) and chlorotype assigned for 337 accessions (Supplementary Table S1). Of the 18 cultivars named Lambrusco or similarly, most are traditionally cultivated in an area between the towns of Parma, Mantova and Modena, mainly in the region of Emilia-Romagna (Fig. 1). The accessions belonging to the subspecies *sylvestris* were distributed in 9 populations (Fig. 1 and Supplementary Table S3). As to plant sex, some vines showed flowers not clearly classifiable as female or hermaphrodite, bearing both gynoecium and partially, not fully developed stamens (Supplementary Table S1).

### Chlorotype analysis

The most frequent chlorotype in the cultivated section was D, covering 76% of the examined variety profiles (Supplementary Figure S1). If we exclude from the dataset the varieties clearly of non-Italian origin (like Cabernets, Merlot, Pinot, etc.), chlorotype D reach 82% of the domesticated compartment. Chlorotype A is also well recurrent in the Italian cultivated germplasm (16%), while C is uncommon (2%) and B very rare in the set of the examined Italian varieties. As for the 65 wild plants, they are divided between chlorotype A, by far the most frequent (77%), and D for the remainder 23% (Supplementary Figure S1).

### Genetic structure of cultivated and wild *vinifera* grapevines

**Figure 2:**
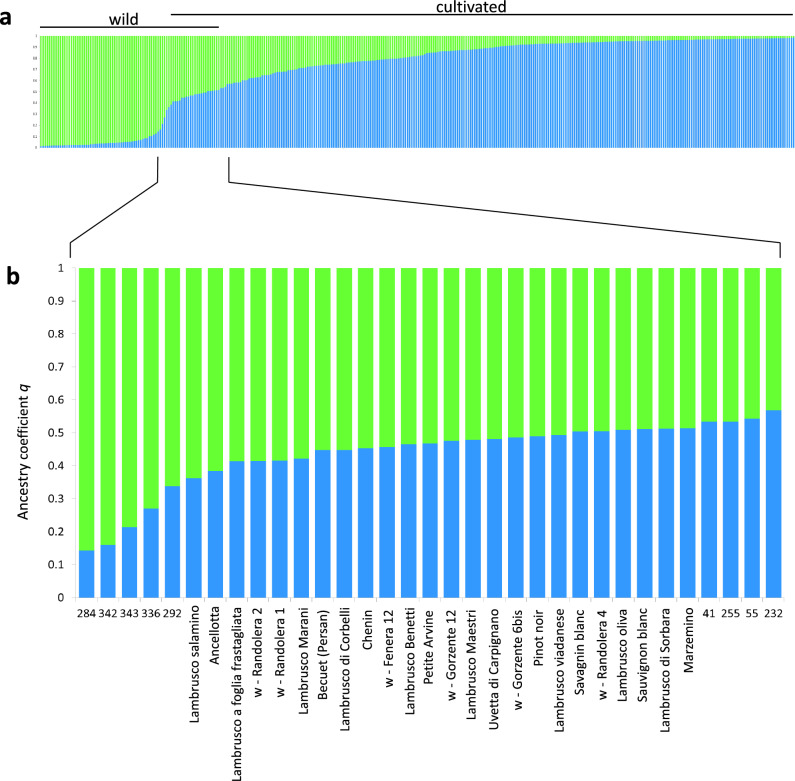
Bar plot obtained by STRUCTURE analysing 348 unique genotypes at 27 nSSR markers. Each genotype is represented by a vertical line divided in coloured segments estimating the ancestry membership proportion to STRUCTURE group 1 and 2 (blue and green for the domesticated and the wild subspecies respectively). Two genetic major groups corresponding to the cultivated and the wild grapevines are well noticeable (**a**). In the enlarged portion of the diagram (**b**) samples with admixed ancestry referred to in the text are labelled. For the other samples the code number according to Supplementary Table S1 is given.

The analysis by STRUCTURE of the 348 non-redundant nSSR genetic profiles suggested the most likely genetic groups were 2, as inferred by the value of Delta-K (Supplementary Figure S2), the two groups consisting in the cultivated and in the wild plants respectively (Fig. 2a). In this model, several samples from the two sections resulted admixed. Assuming a threshold value 0.5 of the membership coefficient (*q*), the proportion of *sylvestris* ancestry was higher than the domesticated in as many as 13 cultivated genotypes out of 283, while it was lower in one individual collected in the wild (Supplementary Table S4). The *q* values of these samples suggested an intermediate ancestry between the two subspecies. They ranged in fact from 0.363 to 0.493 in the group of domesticated for the 13 cultivated genotypes and corresponded to 0.494 in the group of *sylvestris* for the wild plant w-Randolera 4 respectively (Supplementary Table S4).

The 13 varieties genetically closer to the wild populations, ordered by their *q* coefficient values in the group of the domesticated were: Lambrusco salamino, Ancellotta, Lambrusco a foglia frastagliata (Enantio N.), Lambrusco Marani, Becuét (Persan), Lambrusco di Corbelli, Chenin, Lambrusco Benetti, Petite Arvine, Lambrusco Maestri, Uvetta di Carpignano, Pinot, Lambrusco viadanese (Fig. 2b). Among other varieties showing a *q* value in the cultivated group < 0.6, thus with admixed ancestry *sativa-sylvestris*, Savagnin blanc (syn. Traminer), Lambrusco oliva, Sauvignon blanc, Lambrusco di Sorbara, Marzemino had an ancestry coefficient < 0.52 (Fig. 2b and Supplementary Table S4).

On the side of the grapevines collected into the wild, besides w-Randolera 4 (*q* < 0.5 in the wild group), the following 5 plants had a *q* coefficient < 0.6: w-Gorzente 6 bis, w-Gorzente 12, w-Fenera 12, w-Randolera 1 and w-Randolera 2 (Fig. 2b and Supplementary Table S4).

**Figure 3:**
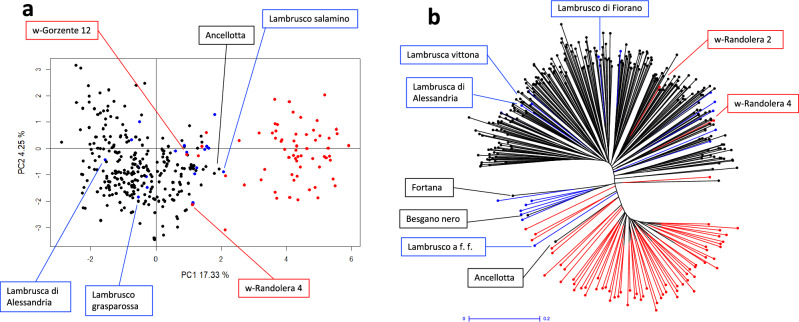
Principal Component Analysis (PCA) diagram plot (**a**) and Neighbour-joining unrooted tree (UwNJ) (**b**) from 348 non-redundant genotypes analysed with 27 nuclear SSR. In both diagrams, samples from the *sylvestris* populations are in red colour, while black and blue are the cultivated varieties and those named Lambrusco respectively. Only cultivated and wild (w-) samples referred to in the text are labelled.

PCA outputs from the analysis of 27 nSSR markers indicated a clear separation of wild plants from cultivars along the axis of the major component explaining 17% of the total variance (Fig. 3a). A pre-calculated principal component analysis generated by balanced ancestral populations (see Materials and Methods for details) produced an output diagram (data not shown) consistent with the projection of all samples showed in Fig. 3a.

Some cultivars including many named Lambrusco resulted genetically close to the *sylvestris* branch together with Ancellotta (a major variety from Emilia-Romagna), while other Lambruscos (or similar), notably Lambrusco grasparossa and Lambrusca di Alessandria, were instead distant. On the other hand, several individuals collected into the wild, like w-Gorzente 12 and w-Randolera 4, were allocated among the cultivated plants or on the border between the two groups.

PCA diagram confirmed the ancestry admixture revealed for several samples by STRUCTURE, spatially describing genetic structure of the examined genotypes.

The UwNJ cluster diagram shows a structure similar to the one from PCA with a clear separation of the wild grapevines mostly gathered at one diagram side and the cultivars distributed in several minor groups at the opposite side (Fig. 3b). Here, again, there are some cultivated varieties clustering inside the *sylvestris* group, with Ancellotta and Lambrusco a foglia frastagliata (Enantio N.) the most genetically close to the wild subdivision as also pointed out by STRUCTURE. A cluster that would appear to be transitional between wild and cultivated grapevines includes 8 cultivars among which 6 Lambruscos, with the addition of Fortana and Besgano nero, all traditional from Emilia-Romagna. Parentage analysis (see specific section of this paper) identified these two local varieties as genitors of 6 Lambruscos. This is explained by the fact that the UwNJ analysis favours kinship relationships in the distribution of the samples. Other Lambrusco (like Lambrusco del pellegrino syn. L. di Fiorano), and varieties similarly named (i.e. Lambrusca vittona and Lambrusca di Alessandria) resulted genetically rather distant or even much distant from the wild plants.

On the other hand, two of the wild individuals clustered inside the cultivars, and some were grouped in a sort of intermediate set between the two subspecies (Fig. 3b).

### Parentage analysis

None of the individuals assumed as wild was found to be a feral accession escaped from cultivation, nor a non-*vinifera* hybrid. This was indicated by both the observations made on plants (leaf and flower morphology) and the analysis of genetic profiles.

Results on the parentage analysis related to the grape cultivars from north-western Italy have been already published by Raimondi et al.^23^. Here we reported the outputs concerning the relationships *sativa-sylvestris* and the cultivated germplasm typical of the region of Emilia-Romagna, from where most of the Lambruscos come as previously mentioned.

Parentage analysis revealed the genotypes collected in the wild showing an admixed ancestry between the two subgroups *vinifera* and *sylvestris* were genetically related to several cultivars. A parent-offspring (PO) link was found between the two individuals w-Randolera 1 and w-Randolera 4 belonging to the wild population Randolera from north-western Piedmont (Fig. 1) and the cultivar Rossara N. (synonyms Schiava N. and Schiava lombarda). The genetic PO relatedness was inferred from high LOD score values and from one shared allele on all the 27 nSSR loci analysed (Table 1 and Supplementary Table S2). Three further nSSR loci analysed were consistent with the mentioned PO (data not shown). The same relationship emerged for plant 2 of the same *sylvestris* population Randolera (w-Randolera 2) and Freisa, an historically widespread variety from Piedmont. Although today, due to the abandonment of grape cultivation in those areas, sporadic familiar vineyards are no less than 2 km distant from the Randolera population site, some decades ago cultivated plots were closer, with a high possibility of contamination of the wild germplasm. With the local name of Uvana we still found in the area isolated, old vines of the cultivar Rossara N. (syn. Schiava), while Freisa is still present mixed up to other varieties in many local longstanding vineyards.

**Table 1:** List of the parent/offspring relationships (PO) resulted from this study between: **(A)** cultivated varieties and wild plants, **(B)** cultivated varieties from the region of Emilia-Romagna, and **(C)** reference PO well documented by literature. Only PO with 0 mismatching loci (except the duo Verdicchio bianco/Lambrusco di Corbelli) and scoring LOD > 11.0 are presented.

Grapevine 1 (Gv1)	Chlorotype Gv1	Grapevine 2 (Gv2)	Chlorotype Gv2	Pairs of nSSR loci compared	Mismatching nSSR loci	Pair LOD score	Literature
**A**							
Rossara N. (Schiava N.)	D	w-Randolera 1	A	27	0	19.6	
Rossara N. (Schiava N.)	D	w-Randolera 4	A	27	0	18.8	
Dolcetto N	D	w-Gorzente 6bis	A	27	0	17.8	
Cagambraga	D	w-Fenera 12	A	27	0	17.6	
Freisa N	D	w-Randolera 2	A	27	0	13.7	
**B**							
Besgano nero	D	Besgano bianco	n.d	27	0	18.2	
Besgano nero	D	Lambrusco Oliva N	A	27	0	17.5	
Besgano nero	D	Lambrusco salamino N	A	27	0	17.1	^19^
Besgano nero	D	Lambrusco Marani N	D	27	0	16.6	^19^
Besgano nero	D	Lambrusco Barghi N	A	27	0	22.4	
Besgano nero	D	Lambrusco Benetti N	A	27	0	14.1	
Coccalona nera (Orsolina, Rohrtraube blauroth)	A	Lambrusco del pellegrino N. (L. di Fiorano)	A	27	0	19.4	^23^
Coccalona nera (Orsolina, Rohrtraube blauroth)	A	Parmesana	D	27	0	18.8	^23^
Coccalona nera (Orsolina, Rohrtraube blauroth)	A	Cavazzina	D	27	0	13.4	
Fortana N	D	Lambrusco Maestri N	A	27	0	23.9	^41^
Fortana N	D	Uva tosca N	D	27	0	12.2	^41^
Rossara N. (Schiava N.)	D	Uvetta (from Carpignano S.)	n.d	27	0	13.5	
Uva tosca N	D	Uva tosca bianca	D	24	0	18.8	^25^
Uva tosca N	D	Lambrusco Montericco N	D	27	0	11.1	^41^
Verdicchio bianco B	D	Lambrusco di Corbelli	D	27	1*	14.8	
**C**							
Cabernet franc N	D	Cabernet sauvignon N	D	27	0	29.7	^42^
Cabernet franc N	D	Merlot N	C	27	0	20.9	^19^
Garganega B	D	Trebbiano toscano B	D	27	0	24.7	^43^
Gouais blanc (Liseiret B.)	C	Gamay N	C	27	0	19.1	^44^
Gouais blanc (Liseiret B.)	C	Riesling B	A	27	0	18.3	^45^
Gouais blanc (Liseiret B.)	C	Chardonnay B	C	27	0	15.0	^44^
Heptakilo	B	Zibibbo B. (Muscat of Alexandria)	B	27	0	30.3	^19^
Moscato bianco B	D	Zibibbo B. (Muscat of Alexandria)	B	27	0	19.7	^46^
Pinot noir N	A	Chardonnay B	C	27	0	24.8	^44^
Pinot noir N	A	Gamay N	C	27	0	15.1	^44^
Sauvignon blanc B	D	Cabernet Sauvignon N	D	27	0	19.1	^42^
Savagnin blanc B	D	Pinot noir N	A	27	0	20.8	^19^
Savagnin blanc B	D	Bian ver B. (Verdesse B)	D	27	0	17.8	^19^
Savagnin blanc B	D	Sauvignon blanc B	D	27	0	15.9	^19^
Savagnin blanc B	D	Chenin B	D	27	0	14.8	^19^
Savagnin blanc B	D	Becuet N. (Persan N)	A	27	0	14.6	^19^

*Mismatching is due to a null allele at locus VvMD36.

The contamination by domesticated forms were observed for wild individuals from two other *sylvestris* populations. The wild grapevine w-Gorzente 6bis, from southern Piedmont, resulted linked to Dolcetto, one of the major Piedmontese variety (Table 1). Fenera plant 12 was PO related with the cultivar Cagambraga, an ancient, today severely endangered variety recently recovered just in one site in north eastern Piedmont. Although this place is a few tens of km away from the wild grapevine population site, it is certainly conceivable that this cultivar, which has now disappeared, was more widespread in the past.

Ultimately, a contaminant specific domesticated variety was found for all the wild genotypes of admixed ancestry according to STRUCTURE.

Coming to the group of the cultivated varieties whose ancestry according to STRUCTURE was shared with the wild subspecies, only in few cases parentage analysis suggested a possible ancestor. As expected, none of the analysed wild individuals show a genetic profile consistent with a parentage likelihood for these varieties, despite their nearly 50% of ancestry attributable to the *sylvestris* genome. This can be easily explained by the fact that the populations of wild grapevines examined today are nothing but a fragmented remnant of the original wild germplasm likely much more expanded and differentiated at the time of the rising of the cited domesticated varieties.

As to the Lambruscos from Emilia-Romagna, Lambrusco Benetti, Barghi, Marani, salamino and oliva were all suggested as offspring of Besgano nero (Table 1), an ancient grape of the same rural areas, likely being a *sylvestris* individual (or a *sylvestris* descendant) the other parent. A possible descendant from a wild plant inter-crossed with Verdicchio bianco (a variety spread from the Italian Adriatic coast to northern Italy) could be Lambrusco di Corbelli.

For other cultivars resulted genetically close to the wild subspecies, PO relationships were confirmed for Becuet (syn. Persan), Bian ver (syn. Verdesse), Chenin, Pinot and Sauvignon as likely descendant from Savagnin, while for other varieties of high *sylvestris* ancestry, like Ancellotta, Lambrusco a foglia frastagliata and Petite Arvine, no possible relative was suggested.

The lineage of other varieties named Lambrusco (or similarly designated) but distant from the *sylvestris* subspecies, like Lambrusca di Alessandria, Lambrusca pignata and Lambruschetta, all from Piedmont, were already examined by^23^. Lambrusco Maestri as likely descendant from Fortana N., and Lambrusco Montericco from Uva tosca confirmed previous findings^25,41^. Coccalona nera (syn. Orsolina, Rohrtraube blaurot) was confirmed one of the major main ancestors in northern Italy.

## Discussion

The distribution of chlorotypes detected in this study confirms what has already been observed in the Italian peninsula^13^ both for *sylvestris* grapevines (with a predominance of the type A and a considerable presence of D) and for cultivars, where the two chlorotypes are predominant but with an inverse ratio (Supplementary Figure S1).

Péros et al.^47^ suggested the species *V. vinifera* arose from the hybridization of two lineages (carrying chlorotypes A and D respectively) expanding from Asia to Europe in separate dispersal events. Types B and C, absent in the Asian species, would develop from A. These findings seem to explain the absence of chlorotype D at the western edge of Europe, and its increasing presence in Europe (together with B and C) from the Italian peninsula moving eastward towards Asia, as well indicated by chlorotype distribution depicted by Arroyo-García et al.^13^.

Unlike other contexts, where it was suggested for some individuals collected in the wild seed dispersal from vineyards^24^, our admixed wild plants shared approximately half of the genome with *sylvestris* subspecies and half with specific varieties. Sangiovese and Trebbiano toscano, leader cultivars in central Italy, were similarly identified as parents of wild accessions in Tuscany^16^.

In the first studies on genetic relationship between the two *vinifera* subspecies, a putative event of secondary domestication has been stated in the Italian island of Sardinia by Grassi et al.^39^. The two involved cultivars Bovale murru and Bovale muristellu (identical to the Spanish varieties Graciano and Parraleta respectively) shared 50% of their alleles at 6 nSSR loci with 3 individuals of a *sylvestris* population. Actually, data from the cited pioneering work indicate more likely an example of wild population contamination by cultivated allochthonous grapevines.

For *sylvestris* populations located in north-western Italy, putative pollen donors were Freisa, Rossara (syn. Schiava or Schiava lombarda) and Cagambraga. The latter two varieties, currently endangered in north western Italy, were recovered in old local vineyards for family use, at the time rather distant from the sites harbouring wild grapevines. Indeed, wild plants can preserve in their descendant generations the evidence of the presence in the past of an intense viticulture now gone. Also, the discover of such pollen donors now rare, was possible thanks to the recovery of the threatened local *sativa* germplasm. As to the admixed wild vines from Gorzente population, the contaminant was likely Dolcetto, one of the major wine grapes of the area. Again, wild plants seem to keep the signs of an impending viticulture for years.

Observations on flower morphology of individuals resulting admixed indicated W-Randolera 4 and w-Gorzente 6 bis showed hermaphrodite flowers, with fully developed stamens and ovary (Supplementary Table S1). In other plants (w-Randolera 1, w-Randolera 2 and w-Fenera 12) a normal ovary with short and straight stamen filaments (instead of revolute as normally in female flowers) were observed. It has not been ascertained whether these flowers were physiologically female or hermaphrodite, although short filaments do not normally go along with anthers developing fertile pollen.

Gene flow from domesticated forms to wild populations had been already reported in different regions and under different ecological conditions^9,11,14,48^. Di Vecchi and collaborators^49^ investigated the process by a paternity-based approach, analysing for their parentage 305 seedlings obtained from female wild plants in different locations. The estimated pollen immigration from the cultivated compartment ranged from 4.6% to as much as 26%, indicating the wild germplasm can be severely affected in its evolution with risk of being denatured and in the long run disappearing. In northern Spain, the wild grapevine populations described suffered a dramatic regression in the 20-year period between prospections, with a significant increase of feral accessions^50^. This is particularly alarming for grapevine genetic diversity preservation. In fact, *sylvestris* forms in compare to the domesticated have been shown to possess genomic regions significantly enriched of functional gene classes related to response to biotic and abiotic stress and thus valuable source of resilience in grapevine breeding programs^51^.

The genetic proximity of several grape cultivars to wild *vinifera* forms, when not explained by the contamination of the latter by the former, indicate events of introgression in the development of local cultivated genetic pool. A genomic approach gave evidence of the presence of an introgression signature in wine varieties across Western Europe and particularly in a subset of Iberian varieties^52^. When the geographic dispersal of a nascent crop to new locations arose, adaptation to local environments is often facilitated by introgression between the crop and locally adapted wild populations^5^. A process of adaptive introgression, although not supported in our study by strict genomic insights, likely occurred in the emergence of several wine grapes examined here.

This seems evident for sought European varieties like Cabernet franc and its descendants (Cabernet sauvignon and Merlot), already found close to the wild genotypes^9,10,16^. As already Bronner^53^ had assumed, in our study Savagnin (syn. Traminer) was confirmed to share an ancestry percentage of around 50% with the *sylvestris* subspecies, with an even higher degree of wild ancestry in its descendants Becuét, Chenin, Pinot and still high in Sauvignon.

Petite Arvine, an ancient reputed wine grape of likely Swiss origin^54^, resulted also highly admixed with the *sylvestris* subspecies. The considerable high vigour of its shoots of long internodes and the berries of reduced size with a minimal yield in must, are all characteristics that cannot failed to recall the forms of wild grapevine.

Coming to Lambruscos (or similarly named cultivars), only those cultivated in the region of Emilia-Romagna (and indeed not all of them) showed a high level of wild ancestry, together with another grape traditional of the same area, Ancellotta.

Therefore, Lambrusca di Alessandria, Lambruca vittona, Lambrusca pignata and Lambruschetta from north western Italy recall in their name the Latin term *lambrusche* for some characters reminding the wild grapevines like the high content of pigments and tannins in their fruits.

An origin by an active process of introgression of genomic regions from the wild compartment appear consistent for Ancellotta and for 9 out of 14 Lambruscos from Emilia-Romagna (including Lambrusco a foglia frastagliata more cultivated in Trentino). In this regard, it appears significant that 4 out of the 9 mentioned Lambruscos exhibit a female flower considered to be an ancestral trait in *vinifera* evolution^40^.

Several Lambruscos, as well as Ancellotta, showed a higher ancestral wild grapevine component among a great set of worldwide spread varieties^6^. Similarly using SNP markers, Lambrusco di Sorbara and L. a foglia frastagliata (Enantio N.) were found genetically close to the wild section also by Magris et al.^12^, while in the same work the other examined Lambrusco (L. grasparossa) was not. Accordingly, our analyses showed Lambrusco grasparossa, L. Barghi, L. Montericco and L. del pellegrino (syn. Lambrusco di Fiorano) had an estimated lower wild ancestry. Despite their name, the origin of this second set of Lambruscos does not seem to directly involve the *sylvestris* subspecies.

As to the emergence of Lambrusco varieties, the fermentation of wild grapes is documented in Emilia-Romagna (the Italian Lambrusco’s region) in XVII century^55^. The transport of wild *lambrusche* grapes, “black and robust”, from Modena and Sorbara to the Ducal cellar to make wine with, is documented in 1693 and 1748^56^.

It is not clear when the term to designate wild plants became the name of cultivated varieties. Historical local chronicles report on a very cold period between 1600 and 1700, leading to the death of many cultivated vines in the region. It is perhaps than that seedlings derived from the contribution of local *sylvestris* began to be used for their rusticity and cold resistance to renew vineyards. What is certain is that Lambruscos as cultivated varieties appeared rather recently in the historical grapevine panorama, generally not before the beginning of XIX century (Table 2). Their names refer to the place where the grape was popular or presumably emerged (Sorbara, Montericco, Fiorano, Viadana), the surname of the person or family to whom the introduction or spreading is owed (Barghi, Benetti, Corbelli, Maestri) or a specific grape feature (grasparossa for the red stem, salamino for the compact long and narrow bunch, oliva from olive for the elongated berries). These specifications arose rather recently, when it became important to distinguish different types within the Lambrusco family.

**Table 2:** The Lambrusco-named varieties and Ancellotta from Emilia-Romagna and Trentino examined in this study.

Variety name	Current surface (hectares on 2022)	First known historical reference		Synonyms/remarks
		Where	When	
Ancellotta	4900	Massenzatico (RE)	Dalla Fossa, 1810	Lancellotta
Lambrusco a foglia frastagliata (Enantio N.)	11	Avio (TN)	Comitato Vitivinicolo della Provincia di Trento, 1954	Enantio
Lambrusco Barghi N. *	4	Albinea (RE)	Early 1800s (Rinaldi and Valli, 1992)	
Lambrusco Benetti N	3	Carpigiano (MO)	Early 1900s (oral communication)	Lambrusco Benatti
Lambrusco del pellegrino N. (L. di Fiorano) *	0.7	Fiorano (MO)	Vicini, 1752	Lambrusco di Fiorano, Lambruscone, Lambrusco oliva grosso
Lambrusco di Corbelli	–	Rivalta (RE)	Pizzi, 1891	Lambrusco di Rivalta
Lambrusco di Sorbara N	1300	Sorbara (MO)	Bertozzi, 1840	Lambrusco dalla viola, Lambrusca
Lambrusco grasparossa N. *	2000	Castelvetro (MO)	Aggazzotti, 1867	
Lambrusco Maestri N	800	Rubiera (RE)	1854 (reported in Sestini and Fabrini, 1862)	Dr. Agostino Maestri (from Rubiera) cultivated and spread this variety
Lambrusco Marani N	490	Plain area of Reggio Emilia province	Pizzi, 1891	Lambrusco Barani
Lambrusco Montericco N.*	19	Montericco (RE)	Pizzi, 1891	Selvatica di Montericco, Lambrusco selvatico, Lambruscone di Montericco
Lambrusco oliva N	200	Sorbara (MO)	Maini, 1851	Olivina nera, Lambrusco mazzone (RE), Olivone (MN), Groppello, Grepello
Lambrusco salamino N	4800	Carpigiano (MO)	Mid 1700s (Paltrinieri P., reported in Bertozzi, 1840)	Salamina
Lambrusco viadanese N	6	Viadana (MN)	Acerbi, 1825	Lambrusco mantovano, Grappello Ruberti

*Lambrusco Barghi, L. del pellegrino, L. grasparossa and L. Montericco showed lower *sylvestris* ancestry. *MN*  Mantova province, *MO* Modena province, *RE* Reggio-Emilia province, *TN* Trento province. Literature references of this table are available in Supplementary Table S6.

The emergence of Lambrusco as cultivated varieties must also deal with the preference of water flooded areas by the western *sylvestris* vines. The historical area of the Lambruscos related to wild *vinifera* are the flooded lands with emerging groundwater around the rivers Po in Emilia-Romagna and Lombardy, and Adige in Trentino (Fig. 1). Until the recent past these sites were neither reclaimed nor sheltered by river banks. The presumed historical landscape of those areas in the centuries between 1500 and 1700 consisted of cultivated fields separated by rows of trees on which domesticated vines grew, interspersed in the more damped sites with remnants of the original forest^57^.

Associating ancestry coefficients with bioclimatic variables, Magris et al.^12^ showed that the genetic component related to western *sylvestris* (wild grapevine from Europe) was significantly and positive associated to seasonal precipitations while the opposite was for the eastern *sylvestris* (wild grapes from the Caucasus regions). These results confirm what reported in pioneering studies on wild *Vitis vinifera* ecology, when it was observed that from the Caucasus towards east the local subspecies, unlike the European *sylvestris,* would prefer arid soils^58^. These observations have found clear confirmation in a recent genomic work on grapevine domestication^6^, where hundreds worldwide samples from the *sylvestris* subspecies clearly clustered in two main groups of European and western Asian origin respectively.

The *sylvestris* subspecies should have introgressed into the new seedlings also genomic regions linked to resistance to bunch rots, whose development is highly facilitated by humid ecological conditions. Among the distinctive regions of the genome between wild and cultivated samples and even within the introgressed tracts, genes connected to the hormonal functionality of plant water regulation and pathogen response have been demonstrated^52^. It is probably no coincidence that most of the Lambruscos exhibit high content in grape anthocyanins and often in tannins. Likely for the same reasons, the French vineyards in the Pyrenean northern slope exposed to Atlantic humid climate harbour native grape varieties with deeply coloured and tannic grapes, such as Tannat and Petit Manseng. These two varieties were both found to be genetically related to the *sylvestris* compartment^10,16^, while Tannat introduced in the humid Uruguayan environment became soon the most popular variety there.

Interestingly, the 4 Lambrusco with less related ancestry to *sylvestris* are varieties mainly developed and still grown in the southern hilly areas of the region, further away from the flooded plain of the Po river (Fig. 1).

As to the Lambrusco’s lineage, the cultivar that most contributed to their origin is Besgano nero (Table 1). Besgano nero is an ancient, at least five century-old grape variety (first mentioned by Gallo^59^), with abundant, juicy berries mainly used for fresh consumption. In the past it was often planted together with trees in single rows dividing the cultivated plots along the middle and lower course of the river Po. Its wide spreading in the plain landscape of the region explains its massive role to the origin of many Lambruscos. Besgano’s chlorotype (D) indicates it was the pollen donor for 4 out of its 5 Lambrusco descendants (Table 1), which shared their type A with by far the most of the *sylvestris* examined plants. Thus, for the 3 Lambruscos (L. oliva, salamino and Benetti), whose wild ancestry resulted around 50%, the state of seedlings collected by farmers at the edges between cultivated fields and forests (if not into the forests) appears consistent.

In conclusion, the cultivars Lambrusco from Emilia-Romagna of admixed genome *sativa-sylvestris*, should be considered examples of a recent process of adaptative introgression from *Vitis vinifera* subsp. *sylvestris* in the cultivated gene pool. This process has made it possible to gain areas for viticulture to which most of the domesticated varieties were not adapted. A greater insight into the functionality of genomic regions involved will explain the selective processes underlying the origin of these Lambruscos. Not proto- or primitive varieties, but examples of active and recent post-domestication.

## Materials and methods

### Plant material

The accession panel (348 non-redundant genotypes, Supplementary Table S1) comprised: (a) a wider group of traditional cultivars (not issued from breeding) from northern Italy and the Italian north central region of Emilia-Romagna, either widely grown or minor varieties and even neglected or at risk of extinction; 18 varieties named Lambrusco (or similarly named) were included in this group; (b) international cultivars among which some like Savagnin blanc, Pinot noir and Cabernet franc already found genetically related to the *sylvestris* subspecies; (c) important traditional cultivars from the Mediterranean area (e.g. Grenache noir, Carignan noir, Muscat à petits grains blancs, Aglianico, etc.) used as outgroup; (d) 65 V. *vinifera* individuals collected in the wild.

Samples consisting in fresh tissue from young shoots (around 2 g) were collected: (a) in the *ex-situ* collection of Grinzane Cavour (http://www.ipsp.cnr.it/en/thematics/turin-headquarter-thematics/grinzane-cavour/) for cultivars from north-western Italy, from the Mediterranean area and international; (b) in commercial vineyards (often old plots) for cultivars from the Emilia-Romagna region, including those named Lambrusco, recovered during a project aimed to the varietal identification of local grape germplasm^60^; (c) in their natural place of discovery for wild grapevines from 9 populations located in northern Italy and southern France (Fig. 1 and Supplementary Table 1). Sample collection was in accordance with the relevant regulations and authorizations. Those from the wild subspecies were collected and identified by authors. Six out of the 9 wild populations were previously inventoried and described^61^. Wild samples from 2 sites in the region of Emilia-Romagna (Pineta di Classe and Pineta San Vitale) were recovered and included in the study because remnants of populations from the same region were the cultivars Lambrusco likely developed. We based our surveys on reports of the presence, albeit sporadic, of *V. vinifera* subsp. *sylvestris* in the Ravenna’s historical pine forests^62^.

### Genotyping

DNA extraction was performed using the protocol described by Thomas et al.^63^, starting from young leaves. All the samples were analysed at 27 nSSR loci (Supplementary Table S5). The first 9 SSR markers were those recommended by the international scientific community and include: the 6 loci selected as genetic descriptors for *Vitis* by the International Organization of Vine and Wine (OIV, https://www.oiv.int/sites/default/files/2022-12/Code%202e%20edition%20Finale.pdf*)* and the 3 markers suggested as common reference for grape variety identification within the European *Vitis* Database (www.eu-vitis.de—“Descriptors/file format—Detailed SSR-marker specific information”). The remaining 18 nSSRs were selected based on their polymorphism and coverage of the 19 chromosomes.

Four chloroplast SSR (cpSSR) markers, sufficient to discriminate the 4 chlorotypes commonly found in *V. vinifera*, were also examined (Supplementary Table S5). The Polymerase Chain Reaction (PCR) conditions used for both nSSRs and cpSSRs are described by Ruffa et al.^64^.

### Ampelographic characterization and trueness-to-type

The examined cultivated grapevines were identified by mean of plant morphology, historical descriptions and their genetic profiles from literature and from the following reference databases: (a) The Italian *Vitis* Database, https://vitisdb.it; (b) VIVC *Vitis* International Variety Catalogue https://www.vivc.de; (c) The Italian Catalogue of grapevine varieties http://catalogoviti.politicheagricole.it/catalogo.php.

All the varieties presumably belong to the *vinifera* species, except for Jacquez, a natural interspecific hybrid included in the analysis because recovered in the key region of Emilia-Romagna, where it was mistaken for a local traditional grape.

All the wild sampled vines were carefully observed and described by expert ampelographers, in order to avoid including, at least as far as revealed by morphology and ecology of the plants, feral individuals escaped from cultivation or hybrids from species other than *vinifera*. Flower sex was also recorded.

### Analyses of population structure

Genetic structure was explored based on the nSSR profiles at 27 loci and using a model-based approach, Principal Component Analysis and hierarchical clustering, as detailed below.

The software STRUCTURE v. 2.3.4^65^ was used to infer the membership of the 348 non-redundant grapevine genotypes to ancestral groups. A number of hypothetical ancestral groups (K) ranging from 1 to 10 was tested, using a Monte-Carlo Markov Chain (MCMC) with 15,000 iterations (10.000 burn-in). To assess the consistency of the results, 10 runs per K value were performed, each one considering an admixture model with uncorrelated allele frequencies among populations. The ΔK criteria implemented in STRUCTURE HARVESTER^66^ v. 0.6.94 (July 2014) was used to estimate the most likely number of genetic groups. Since the most likely K value was 2, STRUCTURE was re-run with a fixed ALPHA value of 0.5 (*i.e.* 1/K) in order to minimize the effect of bias in numerical consistency of the two population subsets, as suggested by Wang^67^.

A Principal Component Analysis (PCA) was focused on estimating admixture between cultivated and wild *vinifera* accessions. To mitigate the bias due to unbalanced ancestral populations, we used a two-step approach consisting in (1) generating principal components (PCs) using balanced ancestral populations, (2) projecting the rest of samples on those pre-calculated PCs (see e.g.^68^). Equal size samples (N = 60) of ancestral cultivated and wild accessions were selected randomly, after excluding Lambrusco-named varieties and putatively admixed accessions based on prior information (varieties previously reported as genetically close to *sylvestris* and wild accessions with flower morphology intermediate between female and hermaphrodite).

Principal components were computed using the *prcomp* function from the *stats* R package (R Core Team, 2022). All the samples not included in ancestral populations were then projected on those PCs. Hierarchical clustering was performed by using the simple matching dissimilarity and the Unweighted Neighbour Joining (UwNJ) method implemented in DARwin v. 6^69^.

### Parentage analysis

Parent–offspring relationships were investigated by a likelihood-based method using the software Cervus v. 3.0.7^70^, accounting for a genotyping error rate of 0.01 in likelihood calculations. For each parent–offspring duos a LOD score (natural log of the overall likelihood ratio) is reported.

### Supplementary Information

Supplementary Figures.

Supplementary Tables.

## Data Availability

All data generated or analysed during this study are included in this published article and its supplementary information files.
