# Supplementary material for: Genetic relationships and introgression events between wild and cultivated grapevines (Vitis vinifera L.): focus on Italian Lambruscos
Source: Sci Rep. 2024 May 29;14:12392. doi: 10.1038/s41598-024-62774-w (PMC11137023; doi:10.1038/s41598-024-62774-w)
Supplement: Supplementary file 1 — Supplementary Figures. [file 41598_2024_62774_MOESM1_ESM.pdf]

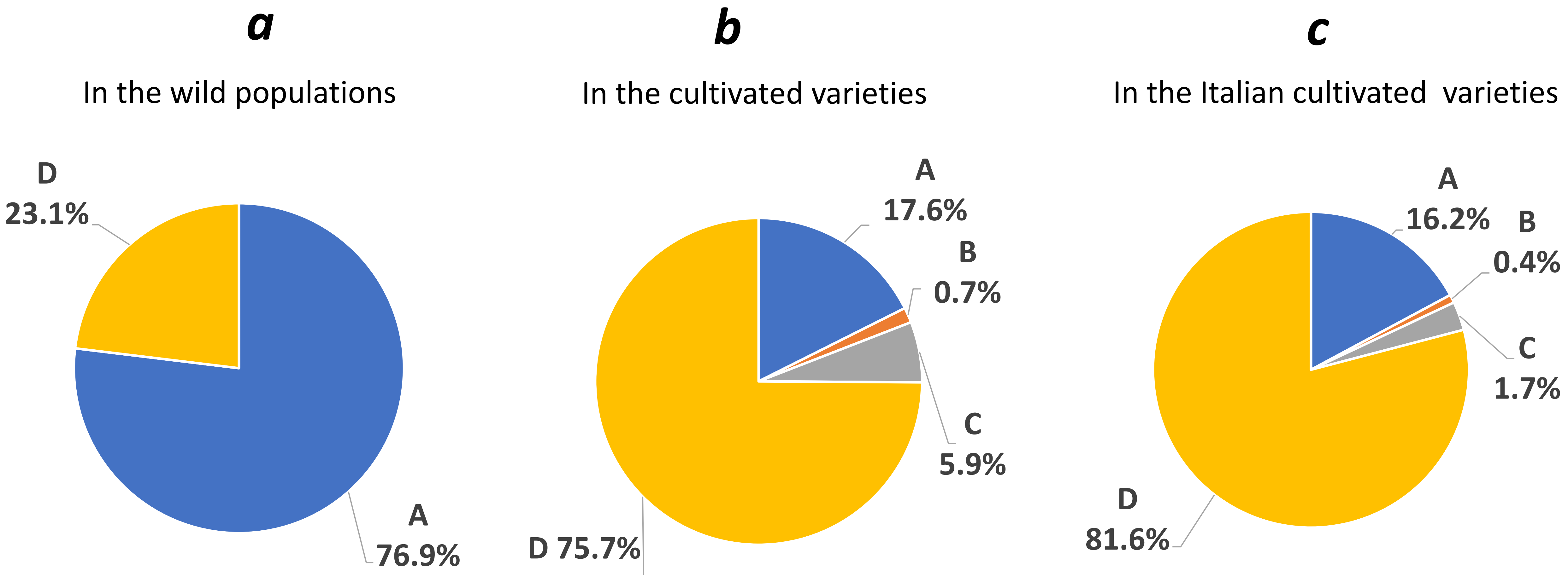

Supplementary Figure S1 – Chlorotype distribution in the examined wild populations (***a***), in the whole set of cultivated varieties (***b***) and in the set of varieties from presumably Italian origin (***c***).

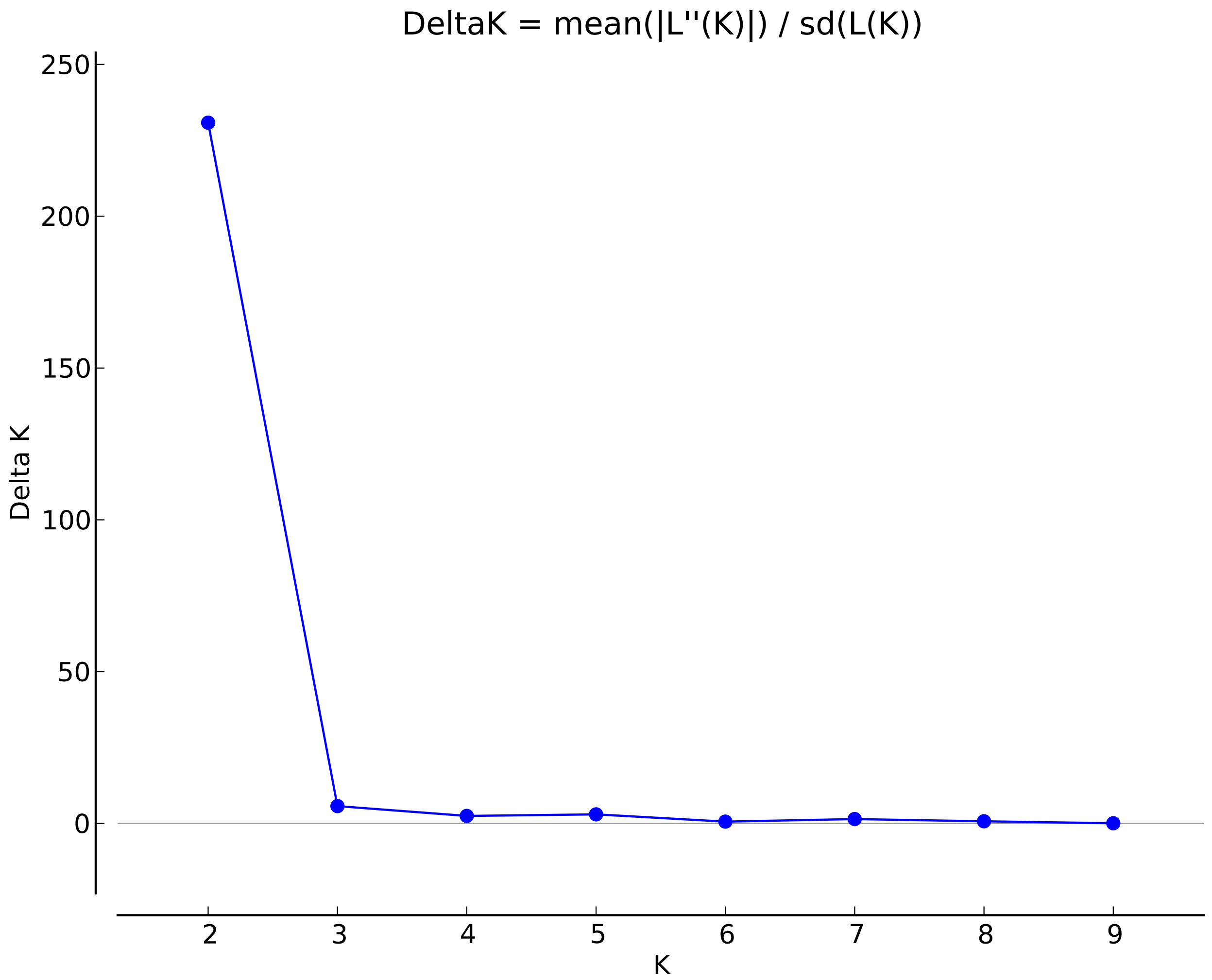

Supplementary Figure S2 – Values of Delta-K plotted by STRUCTURE HARVESTER showing the most likely number of genetic groups identified (K) analysing 348 non–redundant grapevine genotypes.
